# The Evolving Surgical Landscape: A Comprehensive Review of Robotic Versus Laparoscopic Gastrectomy for the Treatment of Gastric Cancer

**DOI:** 10.7759/cureus.49780

**Published:** 2023-12-01

**Authors:** Konstantinos Kossenas, Filippos Georgopoulos

**Affiliations:** 1 Surgery, University of Nicosia Medical School, Nicosia, CYP; 2 Gastroenterology, Al Zahra Hospital Dubai, Dubai, ARE

**Keywords:** chemotherapy, minimal invasive surgery, gastric cancer surgery, research gaps, carcinoma stomach, laparoscopic gastrectomy, robotic gastrectomy

## Abstract

Robotic gastrectomy has been gaining ground in the past 20 years. This study aims to (a) provide an updated and all-encompassing comprehensive review including post-operative outcomes, rate of complications, surgical efficiency and costs, pathology, overall survival, mortality and recurrence, and disease-free survival of robotic versus laparoscopic gastrectomy, (b) report research gaps, and (c) identify ongoing or forthcoming clinical trials that could potentially shed light on underreported findings within the existing literature. Regarding the methodology, PubMed and Google Scholar were searched for randomized controlled trials, systematic reviews, and meta-analyses published between January 2012 and October 2023. ClinicalTrials.gov was searched for related clinical trials currently underway or recruiting. Robotic gastrectomy, when compared to laparoscopic gastrectomy, for the treatment of gastric cancer, performs equally well or shows superiority in terms of the length of hospitalization, overall complications rates, rate of conversion to open surgery, surgical complications, anastomotic leakage, pancreatic complications, blood loss, mortality rates, time to first flatus, time to oral intake, distal and proximal resection margins, recurrence rate, reoperation rates, and overall survival. However, it is associated with higher costs and longer operative time. Parameters such as duodenal stump leakage, anastomosis stenosis, intestinal obstruction, ileus, delayed gastric emptying, wound complications, acute pancreatitis, pancreatic fistula, direct costs, time to initiation of adjuvant chemotherapy, postoperative morbidity, recurrence, and disease-free survival are currently underreported in the literature and necessitate for further research. Lastly, four clinical trials are currently underway or recruiting that could possibly bridge the research gap.

## Introduction and background

Based on data from Global Cancer Observatory (GLOBOCAN) 2020, gastric cancer is the fifth most commonly diagnosed cancer in the world, with an incidence of 1,089,103 cases per year, or 5.6% of all cancer diagnoses. Moreover, gastric cancer accounts for 768,793 deaths per annum, or 7.7% of all cancer-related mortality. Interestingly, Asia accounts for approximately 75% of all gastric cancer diagnoses, 74.8% of all gastric cancer-related deaths, and 77.4% of the total five-year prevalence of gastric cancer in the world [1].

Open gastrectomy with D2 lymphadenectomy has been the staple procedure for gastric cancer resection for many decades before laparoscopy and it is recommended by numerous guidelines [2]. However, advances in minimally invasive surgery, have allowed laparoscopic gastrectomy to be widely accepted as the main curative treatment for gastric cancer. Several clinical trials have shown a decrease in the overall postoperative complication rates in favor of the laparoscopic approach, whilst maintaining equal if not superior oncological outcomes to the open approach [3]. However, laparoscopy still faces drawbacks, such as the limited range of movement, amplification of surgeon’s hand tremors, inconvenient surgical positioning, and two-dimensional (2D) imaging. On the other hand, it is cheaper to implement in daily practice [4].

The leap in surgical technology over the past 20 years has allowed the incorporation of robotic systems in foregut surgery. The utilization of the robotic system aims to provide surgeons with higher-resolution three-dimensional (3D) images, controlled wrist movements, and tremor filtering approaches; aspects that are lacking in laparoscopy [5]. Since the first robotic-assisted gastrectomy reported by Hashizume et al. in 2003, numerous studies have been published showing that the robotic approach may improve short and long-term outcomes, as well as oncological outcomes when compared to laparoscopy [6-8]. However, the evidence is still conflicted on which approach is the superior one and to what extent be incorporated into daily practice. Thus, this study aims to (a) provide an updated and all-encompassing comprehensive review including post-operative outcomes, rate of complications, surgical efficiency and costs, pathology, overall survival, mortality and recurrence, and disease-free survival of robotic versus laparoscopic gastrectomy, (b) report research gaps, and (c ) identify ongoing or forthcoming clinical trials that could potentially shed light on underreported findings within the existing literature.

Methods

Pubmed, Google Scholar, and Scopus were searched for randomized controlled trials (RCTs), systematic reviews, and meta-analyses between 2012 and 2023, which actively compared the robotic to the laparoscopic approach. Keywords related to the subject of interest were used to search the literature, such as: Robotic gastrectomy, laparoscopic gastrectomy, gastric cancer, and systematic review or randomized clinical trial or RCTs or RCT or meta-analysis. For the study to be included in the review, it had to compare the robotic approach directly with the laparoscopic approach, be in English, and either be an RCT or systematic review and/or meta-analysis. Systematic reviews, meta-analyses, and RCTs were chosen as they convey some of the highest levels of evidence. Literature review papers, non-randomized clinical trials, retrospective studies, non-randomized studies, and non-English literature were excluded. 

After removing the duplicates, a total of 31 studies were retrieved; two RCTs and 29 systematic reviews and/or meta-analyses (Table 1). Twenty-six studies were based in China, three in Italy, two in Japan, and one in Portugal. Clinicaltrials.gov was searched to identify clinical trials currently active or recruiting, in which the main or secondary outcomes were to examine parameters currently overlooked in the literature. In total, four trials were identified (Table 2).

**Table 1:** Study characteristics and their key findings arranged in chronological order RG: robotic gastrectomy; WMD: weighted mean difference; LG: laparoscopic gastrectomy; MD: mean difference; RR: risk ratio; HR: hazard ratio

Author	Type of study	Country	Total number of patients	Key findings
Xiong et al., 2012 [9]	Meta-analysis	China	918	Operative time: The RG group had a statistically significant longer operative duration when compared to the LG group (WMD: 68.77, 95%CI: 35.09-102.45; P < 0.0001). Intraoperative blood loss: The RG group had a statistically significant lower blood loss when compared to the LG group (WMD: -41.88, 95%CI: -71.62 to -12.14; P = 0.006). Harvested lymph nodes: No statistically significant difference was observed between the RG and LG groups (WMD: -0.71, 95%CI: -6.78 to 5.36; P = 0.82). Overall morbidity: No statistically significant difference was observed between the RG and LG groups (WMD: 0.74, 95%CI: 0.47 to 1.16; P = 0.19). Perioperative mortality rates: No statistically significant difference was observed between the RG and LG groups (WMD: 1.80, 95%CI: 0.30 to 10.89; P = 0.52) and length of hospital stay: No statistically significant difference was observed between the RG and LG groups (WMD: 0.42, 95 CI: -1.87 to 0.79; P = 0.42)
Xiong et al.,2013 [10]	Meta-analysis	China	2495	Blood loss: The RG group was statistically significantly associated with lower intraoperative blood loss when compared to the LG group. Oral intake: The RG group was statistically significantly associated with a shorter time interval to the first oral intake, when compared to the LG group. Operative time: The RG group was statistically associated with a longer operation time when compared to the LG group. Distal resection margin: The RG group was significantly associated with a shorter distal resection margin when compared to the LG group. No statistically significant difference was observed between the RG and LG groups in the number of harvested lymph nodes, proximal resection margin, rate of conversion to open surgery, overall morbidity, anastomotic leakage, anastomotic stenosis, intestinal obstruction, time to first flatus, duration of hospitalization, and mortality rate
Liao et al., 2013 [11]	Meta-analysis	China	2,235	Operative time was statistically significantly longer in RG when compared to LG (laparoscopic surgery) but with high heterogeneity observed (WMD: -50, 95%CI: -69.93 to -30.07, P < 0.0001, I2 = 88). Blood loss: RG had a statistically significant lower blood loss when compared to LG but with high statistical heterogeneity (WMD: 46.97, 95%CI: 6.12 to 87.83, P < 0.02, I2 = 98). Duration of hospitalization: RG showed a non-statistically significant shorter hospital stay than the LG group, (WMD: 0.50, 95%CI: -0.08 to 1.07, P = 0.09, I2 = 98). Postoperative complication rate: 173 cases of adverse effects occurred out of 1473 patients who underwent LG (11.74%), and 95 cases occurred out of 762 patients who underwent RG (12.46%). However, the results were not statistically significant (OR: 0.88, 95%CI: 0.67 to 1.17, P = 0.38, I2 = 0). Anastomosis leakage: 20 cases occurred in 746 patients who underwent RG (2.68%) and 36 cases occurred in 1425 patients who underwent LG (2.52%). However, there was no statistically significant difference between the RG and the LG groups (OR: 0.92, 95%CI: 0.53 to 1.61, P = 0.78, I2 = 0). Anastomosis stenosis: No statistical significance was observed. LG group: 9 out of 564 patients, RG group: 4 cases out of 310 (OR: 1.26, 95%CI: 0.41 to 3.89, P = 0.69, I2 = 0). Intestinal obstruction rate: The two groups showed no statistically significant differences (OR: 0.66, 95%CI: 0.27 to 1.63, P = 0.37, I2 = 0). Bleeding: No statistically significant difference was observed between the RG and LG groups (OR: 0.70, 95%CI: 0.32 to 1.52, P = 0.37, I2 = 0). No statistically significant difference was observed between the RG and LG groups for proximal resection margin, distal resection margin, harvested lymph nodes, and mortality rate
Hyun et al., 2013 [12]	Systematic review and metanalysis	China	7200	Operative time: RG was associated with a statistically significant longer operating time when compared to the LG group (WMD 61.99; P ≤ 0.001). No statistically significant difference was observed between the RG and LG groups for the number of harvested lymph nodes, resection margins, blood loss, duration of hospitalization, and overall complications.
Shen et al., 2014 [13]	Meta-analysis	China	1,875	Operative time: RG was associated with a longer operative time vs LG (p < 0.05). Estimated blood loss: RG had lower estimated blood loss vs LG (p < 0.05). Distal resection margin: RG had a longer distal margin vs LG (p < 0.05). No statistically significant difference was observed between the RG and LG groups for overall complications, hospital stay, proximal resection margin, and number of harvested lymph nodes.
Chuan et al., 2015 [14]	Meta-analysis	China	1796	Operation time was statistically significantly shorter in the LG group (WMD 42.9; 95%CI 20.87 to 64.92 min; p < 0.05). Blood loss: statistically significantly less in the RG vs the LG group; WMD between RG and LG groups was -16.07 (95%CI -32.78 to 0.64 mL; p < 0.05). Duration of hospitalization: statistically significantly shorter in the RG vs the LG group; WMD between RG and LG groups was -1.98 (95%CI -3.66 to -0.3 days; p < 0.05). No statistically significant difference was observed between the RG and LG groups for harvested lymph nodes, resection margins, and postoperative complications.
Hu et al., 2016 [15]	Meta-analysis	China	3,580	Blood loss: RG was statistically associated with less blood loss when compared to the LG group. Time to first flatus: RG was statistically associated with earlier onset of first flatus when compared to the LG group. Harvested lymph nodes: the RG group was statistically associated with a greater number of harvested lymph nodes when compared to the LG group. Duration of hospitalization: The RG group was statistically associated with a shorter hospital stay when compared to the LG group. Operative time: the RG group was associated with a longer operation duration when compared to the LG group. No statistically significant difference was observed between the RG and LG groups in terms of overall complications, mortality rate, conversion to open surgery, and distal and proximal resection margins.
Wang et al., 2017 [16]	Systematic review and meta-analysis	China	562	Operative Time: one study showed that the operative time was statistically significantly longer in the RG vs the LG group with a WMD of 21.49 minutes (95%CI: 12.48-30.50), (P < 0.00001). No statistically significant difference was observed between the RG and LG groups in terms of estimated blood loss, harvested lymph nodes, overall complications, and duration of hospitalization.
Duan et al., 2017 [17]	Meta-analysis	China	3503	Blood Loss: RG had a smaller blood loss with the WMD between RG and LG being -36.50, with a 95%CI from -61.39 to -11.61. Time to Oral Intake: The WMD for the RG vs the LG group was -0.28, with a 95%CI ranging from -0.46 to -0.09, suggesting a shorter time to initiate oral intake in RG. Operative Time: RG had a longer operative time in comparison to LG. The WMD was 53.48, with a 95%CI ranging from 38.84 to 68.12. No statistically significant difference was observed between the RG and LG groups in terms of postoperative flatus, length of hospital stay, overall postoperative complications, and number of lymph nodes harvested.
Pan et al., 2017 [18]	Meta-analysis	China	1614	No statistically significant difference was observed between the RG and LG groups for overall survival, disease-free survival, and recurrence rate.
Wang et al., 2017 [19]	Systematic review and meta-analysis	China	3744	Operation time: significantly shorter in the LG group vs the RG group; WMD 42.0 (95% CI 28.11-55.89) minutes; P < .00001], Blood loss: was lower in the RG vs the LG group (P = .01). Harvested lymph nodes: No statistical significant difference was observed between the RG and LG groups for duration of hospitalization, proximal resection margin, distal resection margin and postoperative complications.
Guerra et al., 2018 [20]	Systematic review and meta-analysis	Italy	2026	Pancreatic morbidity: RG group: 1.7% (10 out of 570 patients) LG group: 2.5% (36 out of 1456 patients), no statistically significant: odds ratio (OR) of 0.8 [0.31, 2.04], Z = 0.47, P = 0.64. Postop acute pancreatitis: RG group: 0.6% LG group: 0.5% no statistically significant differences (OR= 1.24, Z = 0.32, P = 0.75). Postoperative pancreatic fistula (POPF): 3.5% of patients in total, RG group: 2.7% LG group: 3.8% no statistically significant difference OR: 0.72 [0.15, 3.56], based on data from 1089 patients from four studies (Z = 0.40, P = 0.69). Operative time: RG group: between 206 and 439 minutes LG group: between 167 and 361 minutes The mean difference (MD) was 59.44 [37.92, 80.96], with statistical significance (Z = 5.41, P < .00001). Mean length of hospital stay ranged from 6 to 15 days. RG group vs LG, MD of -0.19 [-1.26, 0.87] (Z = 0.36, P = .72). but without statistical significance. Mean number of harvested lymph nodes ranged between 25 and 44 globally. There was a statistically significant difference favoring RG vs LG, with an MD of 2.92 [0.32, 5.53], (Z = 2.2, P = 0.03).
Bobo et al., 2019 [7]	Meta-analysis	China	4576	Operative Time: RG had a statistically significant longer operative time when compared to LG; mean difference (MD) of 57.98 minutes (P < 0.00001). Blood Loss: RG had statistically significantly less blood loss when compared to LG, with an MD of -23.71mls (P = 0.005). Time to First flatus: RG had a statistically significant shorter time to first post-operative flatus passed when compared to LG, with an MD of -0.14 days (P = 0.03). No statistically significant difference was observed between the RG and LG groups in terms of harvested Lymph Nodes, overall complications, reoperation rate, mortality rate, rate of conversion to open surgery, and proximal and distal resection margin.
Liao et al., 2019 [21]	Meta-analysis	China	3410	Overall survival: No statistically significant difference was observed between the RG and LG groups; OS (HR, 0.98; 95%CI, 0.80–1.20; P = 0.81), (I2 = 0, P = 0.94). Disease-free survival: No statistically significant difference was observed between the RG and LG groups (HR = 1.08; 95%CI, 0.26–4.44; P = 0.67). Recurrence-free survival: No statistically significant difference was observed between the RG and LG groups (HR, 0.92; 95% CI, 0.72–1.19; P = 0.53), (I2 = 0%, P = 0.97). Recurrence rate: RG group: 12.63% (109/863) LG group: 13.30% (255/1917). No statistically significant difference was observed between the RG and LG groups (OR, 0.92; 95% CI, 0.71–1.19; P = 0.53), (I2 = 0, P = 0.71). Length of hospitalization: No statistically significant difference was observed between the RG and LG groups in the Length of hospitalization with an MD of -0.24. The 95%CI for length of hospitalization ranged from -0.60 to 0.11. p-value = 0.16. Postoperative complications: No statistically significant differences were observed between the RG and LG groups with an OR of 0.90 (95%CI: 0.72 to 1.12). p= 0.34. 30-day mortality rate: No statistically significant difference was observed between the RG and LG groups with a risk difference of 0.01, 95%CI 0.00 - 0.01, p= 0.19. Conversion to open surgery: No statistically significant difference was observed between the RG and LG groups with a risk difference of 0.00. 95%CI -0.01 to 0.01, p=0.67.
Qiu et al., 2019 [22]	Meta-analysis	China	8,413	Operative Time: The RG had a statistically significant longer operative time when compared to LG. The WMD was 44.11 minutes, 95%CI ranged from 24.20 to 64.01 minutes, p< 0.0001, I2 = 99%. Intraoperative Blood Loss: The RG group had a statistically significantly lower intraoperative blood loss when compared to LG. The WMD was -17.78 mls, 95%CI ranged from -25.62 to -9.94 mls, p-< 0.00001, I2 = 89%. No statistically significant difference was observed between the RG and LG groups regarding time to first flatus, conversion rate, and mortality rate.
Ma et al., 2020 [23]	Systematic review and metanalysis	China	7275	Operative time: the RG group was associated with a statistically significant longer operative time when compared to the LG group, with a WMD of -32.96. 95%CI for operative time ranged from -42.08 to -23.84, p<0.001. Blood loss: RG resulted in statistically significantly less blood loss when compared to the LG group, with a WMD of 28.66. 95%CI ranged from 18.59 to 38.7, p<0.001. Time to first flatus: statistically significantly shorter for the RG group when compared to the LG group, with a WMD of 0.16, 95%CI ranging from 0.06 to 0.27, p=0.003. No statistically significant difference was observed between the RG and LG groups for the duration of hospitalization, overall postoperative complications, mortality, number of harvested lymph nodes, proximal resection margin, distal resection margin, overall survival, recurrence-free survival, and recurrence rate
Ojima et al., 2021 [3]	Randomized clinical trial	Japan	241	Intra-abdominal infections: no statistically significant difference was observed between the RG and the LG; the incidence rates were 6.2% in the RG group and 8.5% in the LG group. Conversion to open surgery: There was no statistically significant difference in the conversion rates between the groups. LG group: 1.6% (two out of 122 patients). RG group: 3.4% (four out of 119 patients). Postoperative complications of grade II or higher: LG group (19.7%) vs the RG group (8.8%). The difference was statistically significant: p-value of 0.02. Complications limited to grade IIIa or higher: LG group had 16.2% vs the RG group with a 5.3%: p-value of 0.01.
Guerrini et al., 2021 [24]	Meta-analysis	Italy	17,712	Operative time: 267.34 mins in the RG group vs 220.48 min in the LG group. A statistically significant difference was observed (MD 44.73, 95%CI 36.01, 53.45, p < 0.0001). Blood loss: statistically significantly higher estimated blood loss in the LG group vs the RG group (MD -18.24, 95%CI -25.21, −11.26, p < 0.0001). The mean intra-operative blood loss in the RG group was 98.77 cc vs 115.02 cc in the LG group. Conversion to open surgery: RG group: 0.53% (20/3777), LG group: 0.7% (67/9584). No statistically significant difference (OR 0.76, 95%CI 0.45, 1.28, p = 0.30). Reoperation rate: RG group: 1.29% (25/1939), LG group: 1.23% (55/4467). No statistically significant difference (OR 1.01, 95%CI 0.60, 1.68, p = 0.98). Mortality rate: RG group: 0.36% (16/4378) LG group: 0.30% (31/10354) no statistically significant difference (OR 1.43, 95%CI 0.77, 2.65, p = 0.25). Length of hospital stay: RG group: 8.67 days LG group: 9.29 days no statistically significant difference (MD -0.32, 95%CI -0.71, 0.07, p < 0.11). Time to first flatus: RG group: 3.08 days LG group: 3.30 days no statistically significant difference (MD -0.19, 95%CI -0.45, 0.07, p < 0.16). Time to oral intake: RG group: 4.25 days LG group: 4.43 days. The difference observed was statistically significant (MD -0.20, 95%CI -0.30, −0.10, p = 0.0001). Overall complications: RG group: 12.75% LG group: 13.62%, no statistically significant difference (OR 0.91, 95%CI 0.79, 1.04, p = 0.16). Surgical complications (Clavien-Dindo classification): RG group: 4.13% LG group: 6.44% statistically significant difference (OR 0.66, 95%CI 0.49, 0.88, p = 0.005). Anastomotic leakage: RG group: 1.88% LG group: 1.94% No statistically significant difference (OR 1.04, 95%CI 0.79, 1.37, p = 0.78). Pancreatic complication: RG group: 0.81% LG group: 1.28% not a statistically significant difference (OR 0.73, 95%CI 0.43, 1.23, p = 0.24). Cost: RG group average total costs: $12,224.54, LG group average costs $8,292.78 statistically significant difference (MD $3,912.98, 95%CI $3,061.53, $4,764.42, p < 0.00001). Distal resection margin: RG group: 6.70 cm LG group: 6.49 cm no statistically significant difference [MD 0.27, 95%CI -0.15, 0.69, p = 0.21). Proximal resection margin: RG group: 4.46 cm LG group: 4.35 cm no statistically significant difference (MD 0.01, 95%CI -0.14, 0.17, p = 0.87). Recurrence rate: RG group: 9.9% LG group: 13.5% not a statistically significant difference (OR 0.86, 95%CI 0.67, 1.11, p = 0.25). Number of retrieved lymph nodes: RG group’s retrieved lymph nodes mean: 36.63, LG group’s retrieved lymph nodes mean 35.11 statistically significant difference (MD 1.84, 95%CI 0.84, 2.84, p = 0.0003). Modified intention-to-treat analysis (all p < 0.001): RG patients had lower inflammatory responses, faster postoperative recovery, and reduced morbidity vs LG (9.2% vs. 17.6%, respectively, p = 0.039). RG retrieved more lymph nodes whilst maintaining lower noncompliance vs LG. RG had earlier initiation of adjuvant chemotherapy vs LG. RG had higher total hospital costs vs LD. RG had lower direct costs than LD
Lu et al., 2021 [25]	RCT	China	283	RG group exhibited: Faster postop. recovery, milder inflammatory responses, and reduced postop morbidity (9.2% vs. 17.6%, P = 0.039). Number of lymph nodes retrieved: RG extraperigastric lymph nodes: 17.6 ± 5.8 vs. LG: 15.8 ± 6.6, P = 0.018). Noncompliance rate RG: 7.7% vs. 16.9% for LG, P = 0.006). RG group initiated adjuvant chemotherapy earlier: Median (interquartile range) postoperative days: 28 (24-32) vs. 32 (26-42) for LG, P = 0.003. Costs: Total hospital costs were higher in the RG vs LG. Direct cost was lower for RG vs LG (all P < 0.001).
Feng et al., 2021 [26]	Systematic review and meta-analysis	China	13,446	No statistically significant difference was observed between the RG and LG groups for proximal resection margin, distal resection margin, abdominal bleeding, Ileus, anastomosis leakage, duodenal stump leakage, rate conversion to open surgery, reoperation rate, overall survival rate, and recurrence-free survival rate. Operative time; RG group had a statistically significantly longer operative time when compared to the LG group (p < 0.00001). Blood loss: RG group had a statistically significant lower blood loss when compared to the LG group (p <0.0001). Time to first flatus: RG group had a statistically significant earlier time to first flatus when compared to the LG group (p = 0.0003). Time to oral intake: The RG group had a statistically significant earlier time to oral intake when compared to the LG group (p = 0.0001). Duration of hospitalization: The RG group had a statistically significant shorter duration of hospitalization when compared to the LG group (p = 0.0001). Major complications: The RG group had statistically significantly fewer major complications when compared to the LG group (p = 0.0001), Overall complications: The RG group had statistically significantly fewer overall complications when compared to the LG group (p = 0.0003). Harvested lymph nodes: The RG group had statistically significantly more harvested lymph nodes when compared to the LG groups (P < 0.0001). Costs: The RG group had statistically significantly higher costs when compared to the LG group (p < 0.00001).
Zhang et al., 2021 [27]	Meta-analysis	China	3293	Blood loss: RG had statistically significant lower blood loss when compared to LG group. Harvested lymph nodes: RG group had a statistically significant higher number of harvested lymph nodes when compared to the LG group. Time to first oral intake: RG group had a statistically significant shortened time for first oral intake when compared to the LG group Duration of hospitalization RG group had a statistically significant decreased duration of hospitalization when compared to the LG group. Distal resection margin: RG group had a statistically significant longer length of distal resection margin when compared to the LG group. Operative time: RG had a statistically significant increased operative duration when compared to the LG group. No statistically significant difference was observed between the RG and LG groups regarding first postoperative anal exhaust time, proximal resection margin, overall postoperative complication rate, anastomotic leakage, postoperative gastric emptying disorder, pancreatic fistula rate, recurrence rate, and mortality rate.
Wu et al., 2021 [28]	Meta-analysis	China	4142	Overall survival: No statistically significant difference was observed in the OS between the RG and LG groups (HR = 0.97, 95%CI = 0.80-1.19, P = 0.80). Disease-free survival: No statistically significant difference in DFS was observed between the RG and LG group (HR = 0.94, 95%CI = 0.72-1.23, P = 0.65). Three-year OS: No statistically significant difference was observed between the RG and LG groups. No statistically significant difference was observed between the RG and LG groups regarding 5-year OS, 3-year DFS, 5-year DFS, and recurrence rate (OR = 0.88, 95%CI = 0.69-1.12, P = 0.31).
Zizzo et al., 2022 [5]	Systematic review	Italy	3176 to 17,712 in all studies	Operative time: It was longer in the RG group vs the LG group. This result was statistically significant in all seven studies. Blood loss: Lower in the RG group vs LG group this result was statistically significant in all seven studies. Conversion to open surgery: Three out of seven studies reported the conversion to open surgery, however, no statistically significant differences were observed between RG and LG groups Number of lymph nodes retrieved: In five out of the seven studies, the RG group had a greater number of lymph nodes retrieved when compared to the LG group. The difference was also statistically significant. Proximal resection margins: No statistically significant differences were observed between the RG and LG groups Distal resection margins: two out of the six included studies showed a statistically significant difference between the RG group in one study and the LG group in another one. Time to first flatus: Statistically significantly shorter in the RG group in four out of five studies. Time to restart oral intake: statistically significantly shorter in the RG group in four out of five studies. Length of hospitalization: three out of six studies concluded statistically significant shorter stays in the RG group compared to the LG group. Overall complications: In three out of seven studies, the overall complication rates were statistically significantly lower in the RG group than in the LG group. CD ≥ III complication rates were statistically significantly lower in the RG group vs the LG group in three out of four studies. Mortality: No statistically significant differences in mortality rates between RG and LG. Pancreatic complications: RG group had statistically significantly lower rates of pancreatic complications vs the LG group in two out of four studies. Reoperation rates: No statistically significant differences between RG and LG groups. Anastomotic leakage: No statistically significant differences between RG and LG groups. Delayed gastric emptying: No statistically significant differences between RG and LG groups. Long-Term Outcomes: No statistically significant differences were observed between RG and LG groups regarding recurrence-free survival, recurrence rates, and overall survival. Costs: Costs were statistically significantly higher in the RG vs the LG groups, in three out of seven studies.
Jin et al., 2022 [29]	Meta-analysis	China	12,401	Postoperative complications: RG patients had statistically significantly fewer postop. complications compared to LG: OR of 0.81 (95%CI 0.71-0.93; P = 0.002). Pancreas-related complications were statistically significantly lower in the RG group (OR 0.376; 95%CI 0.156-0.911; P = 0.030). Harvested Lymph Nodes: RG had a statistically significant higher number of harvested lymph nodes vs LG (WMD 2.03; 95%CI 0.95-3.10; P < 0.001). Time to First Flatus: RG patients had a statistically significant earlier time of first flatus when compared to LG (WMD -0.105 days; 95%CI -0.207 to -0.003; P = 0.044). Operation Time: RG had a statistically significant longer operation time when compared to LG; WMD of 40.192 minutes (95%CI 32.07-48.31; P < 0.001). Blood Loss: RG group had statistically significantly less blood loss when compared to LG; WMD of -20.09 ml (95% CI -26.86 to -13.32; P < 0.001). Costs: RG was associated with statistically significant higher costs when compared to LG; WMD of 19,141.68 RMB (Chinese Yuan) (95%CI 11,856.07-26,427.29; P < 0.001). Three-year overall survival: no statistically significant differences between RG and LG (OR 1.030; 95%CI 0.784-1.353; P = 0.832). Five-year overall survival: No statistically significant differences between RG and LG (OR 0.862; 95%CI 0.721-1.031; P = 0.105). Conversion rate to open surgery: No statistically significant differences between RG and LG (OR 0.857; 95%CI 0.443-1.661; P = 0.648). Postoperative hospital stay: No statistically significant differences between RG and LG (WMD -0.368 days; 95%CI -0.75-0.013; P = 0.059). Mortality: No statistically significant differences between RG and LG (OR 1.248; 95%CI 0.514-3.209; P = 0.592). Reoperation rate: No statistically significant differences between RG and LG (OR 0.855; 95%CI 0.479-1.525; P = 0.595).
Sun et al., 2022 [30]	Meta-analysis	China	2763	Operative time: RG group had a statistically significant longer operative time when compared to the LG group (MD = 31.42, 95% CI [22.88, 39.96], p < 0.00001). Blood loss: the RG group had statistically significantly less blood loss when compared to the LG group (MD = - 25.89, 95%CI - 36.18, - 15.6, p < 0.00001). Harvested lymph nodes: The RG group had a statistically significantly higher number of harvested lymph nodes when compared to the LG group (MD = 3.46, 95%CI 2.94, 3.98, p < 0.00001). Time to first flatus: the RG group had a statistically significant shorter time to first flatus when compared to the LG group (MD = - 0.08, 95%CI - 0.13, - 0.02, p = 0.006). Time to first liquid intake: the RG group had a statistically significant earlier first liquid intake when compared to the LG group (MD = - 0.13, 95%CI - 0.22, - 0.05, p = 0.002). No statistically significant difference was observed between the RG and LG groups regarding time to start soft diet, postoperative hospital stays, overall complications, complications Grade I-II, complications Grade ≥ III, anastomotic leakage, bleeding, intra-abdominal bleeding, intraluminal bleeding, Ileus, abdominal infection, delayed gastric emptying, and wound complications.
Gong et al., 2022 [31]	Systematic review and meta-analysis	China	5386	Operating time: RG was associated with a statistically significantly longer operative time when compared to the LG group (MD = 43.88, 95%CI = 35.17-52.60). Intraoperative blood loss: RG was associated with statistically significantly less intraoperative blood loss when compared to the LG group (MD = - 24.84, 95%CI = - 41.26 to - 8.43). Number of harvested lymph nodes: RG was associated with a statistically significantly higher number of harvested lymph nodes when compared to the LG group (MD = 2.41, 95%CI = 0.77-4.05). Time to first flatus: RG was associated with a statistically significant shorter time to first flatus when compared to the LG group (MD = - 0.09, 95%CI = - 0.15 to - 0.03). However, in the propensity-score-matched meta-analysis, the difference became non-significant. Length of hospital stay: RG was associated with a statistically significant shorter length of hospitalization when compared to the LG group (MD = - 0.68, 95%CI = - 1.27 to - 0.08). However, in the propensity-score-matched meta-analysis, the difference becomes non-significant. Incidence of pancreatic fistula: RG was associated with a statistically significant lower incidence of pancreatic fistula when compared to the LG group (OR = 0.23, 95%CI = 0.07-0.79). No statistically significant difference was observed between the RG and LG groups regarding proximal resection margin, distal resection margin, time to start liquid and soft diets, and overall complications
Baral et al., 2022 [32]	Meta-analysis	China	20,151	Operative time: RG group had a statistically significant longer operative time when compared to the LG group (WMD = 35.72, 95%CI = 28.59-42.86, P < 0.05) (RG: mean ± SD = 258.69 min ± 32.98; LG: mean ± SD = 221.85 min ± 31.18). Blood loss: RG group had statistically significantly less blood loss when compared to the LG group: (WMD = -21.93, 95%CI = -28.94 to -14.91, P < 0.05) (RG: mean ± SD = 105.22 ml ± 62.79; LG: mean ± SD = 127.34 ml ± 79.62). Number of harvested lymph nodes: the RG group had a statistically significant higher number of harvested lymph nodes when compared to the LG: (WMD = 2.81, 95% CI = 1.99-3.63, P < 0.05) (RG: mean ± SD = 35.88 ± 4.14; LG: mean ± SD = 32.73 ± 4.67). Time to first postoperative food intake: RG group had a statistically significant shortened time to first post-operative food intake when compared to the LG group (WMD = -0.20, 95% CI = -0.29 to -0.10, P < 0.05) (RG: mean ± SD = 4.5 d ± 1.94; LG: mean ± SD = 4.7 d ± 1.54). Length of hospitalization: The RG group had a statistically significant shorter duration of hospitalization when compared to the LG group (WMD = -0.54, 95%CI = -0.83 to -0.24, P < 0.05) (RG: mean ± SD = 8.91 d ± 6.13; LG: mean ± SD = 9.61 d ± 7.74). No statistically significant difference was observed between the RG and LG groups regarding time to first postoperative flatus, overall postoperative complications, proximal resection margin, distal resection margin, mortality rate, conversion rate, and 3-year overall survival rate.
Ali et al., 2022 [33]	Systematic review and meta-analysis	China	13,585	Blood loss: RG group had statistically significantly less blood loss when compared to the LG group: (MD = -17.97, 95% Cl: -25.61 to 10.32, p < 0.001). Clavien-Dindo grade Ⅲ: RG group had statistically significant lower CDIII grade complications when compared to the LG group: (OR = 0.60, 95%CI: 0.48-0.76, p < 0.01). Harvested lymph nodes: RG group had a statistically significantly higher number of harvested lymph nodes when compared to the LG group: (MD = 2.62, 95%CI: 2.14-3.11, p < 0.001). No statistically significant difference was observed between the RG and LG groups regarding distal resection margin, proximal resection margin, conversion rate, anastomotic leakage, and overall complications
Yu et al., 2023 [8]	Systematic reviews and meta-analysis	China	718	Operative Time: RG had a statistically significantly longer operative time when compared to LG, with an MD of 28.20 minutes (P < 0.00001). However, high heterogeneity (I2 = 91%) was observed. Blood loss: RG group had a lower blood loss compared to LG, with an MD of 0.28 milliliters (P = 0.99). However, there was high heterogeneity (I2 = 84%) and the results were not statistically significant. Number of Retrieved Lymph Nodes: There was no statistically significant difference between RG and LG, with an MD of 1.50 (P = 0.54). High heterogeneity was also observed (I2 = 76%). Overall Complications: The rate of overall complications was lower for RG when compared to LG, this difference was not statistically significant, with an OR of 0.99 (P = 0.98). However, low heterogeneity was observed (I2 = 0%). Time to First Flatus: RG had a statistically significant shorter time to the first flatus when compared to LG, with an OR of -0.46 (P < 0.00001). Moderate heterogeneity was observed (I2 = 49%). Time to Oral Intake: Statistically significant earlier onset of oral intake for RG than LG, with an OR of -0.46 (P = 0.0010). Low heterogeneity was observed (I2 = 0%). Length of Hospital Stay: RG had a statistically significant shorter length of hospital stay when compared to LG, with an MD of -0.81 days (P = 0.0002). Also, low heterogeneity was observed (I2 = 0%)
Loureiro et al., 2023 [34]	Systematic review and metanalysis	Portugal	25,521	No statistically significant difference was observed between the RG and LG groups regarding the conversion rate, reoperation rate, mortality, overall complications, anastomotic leakage distal resection margin, proximal resection margin, and recurrence rate. Blood loss: The RG group had statistically significantly less blood loss when compared to the LG group, (MD -19.43 mL, P < .00001). Duration of hospitalization: The RG group had a statistically significant lesser duration of hospitalization when compared to the LG group (MD -0.50 days, P = .0007). Time to first flatus: The RG group had a statistically significant earlier onset of first flatus when compared to the LG group (MD -0.52 days, P < .00001). Time to oral intake: The RG group had a statistically significant earlier onset of oral intake when compared to the LG group (MD -0.17 days, P = .0001). Clavien-Dindo grade ≥ III: The RG group had a statistically significant lower complication rate when compared to the LG group (RR 0.68, P < .0001). Pancreatic complications: The RG group had statistically significantly fewer pancreatic complications when compared to the LG group (RR 0.51, P = .007). Harvested lymph nodes: The RG group had a statistically significantly higher number of harvested lymph nodes when compared to the LG group. Operative time: The RG group had a statistically significant longer operative time when compared to the LG group (MD 41.19 minutes, P < .00001). Costs: The RG group had statistically significant increased costs when compared to the LG group (MD $3684.27, P < .00001).
Shibasaki et al., 2023 [35]	Systematic review	Japan	n/a	Intraoperative blood loss volume: RG has lower intraoperative blood loss volume than LG. Length of hospital stay: the RG group had a shorter length of hospitalization. Mortality: both procedures had similar mortality. Operative time: the RG group had longer operative time when compared to the LG group. Costs: The RG group had higher costs when compared to the LG group

**Table 2:** Clinical trials currently investigating the outcomes of RG vs LG RG: robotic gastrectomy; LG: laparoscopic gastrectomy; CRP: C-reactive protien

Study title and country	Type of study	Aim	Study design	Primary outcomes	Secondary outcomes
“Study of the Clinical Efficacy of Robotic and Laparoscopic Gastrectomy in Neoadjuvant Gastric Cancer (CLASS14)” NCT06042998 [36]; Qingdao University; Country: China; Expected year of completion: 2027	Prospective, multicenter, randomized, controlled study	To investigate the clinical efficacy of robotic radical gastrectomy vs laparoscopic radical gastrectomy, in patients with gastric adenocarcinoma (cT2N+M0 or cT3-4a/N+M0, phase II and III), undergoing neoadjuvant treatment	294 patients in each group, Experimental group: RG, Comparator: LG	Three-year disease-free survival	Three-year overall survival rate, 30-day overall postoperative morbidity rate, 14-day postoperative recovery course
“Robotic, Laparoscopic and Open Gastrectomy Compared on Short and Long Term Outcomes” (IMIGASTRICII); Amilcare Parisi, International Study Group on Minimally Invasive Surgery for Gastric Cancer (Responsible Party) (NCT02751086) [37]; Expected year of completion: 2024; Country: Italy	Prospective, Observational, Multicenter Study	To create and maintain a multi-institutional database comprising information regarding clinical, surgical, and oncological features of patients that will undergo robotic, laparoscopic, or open surgery for gastric cancer and subsequent follow-up.	5000 patients; Experimental group: RG, comparator: LG, comparator: open gastrectomy	Rate of patients with intraoperative adverse effects: during surgery, mean number of harvested lymph nodes, 1-year mortality, 2-year mortality, 3-year mortality, 4-year mortality, 5-year mortality,	Mean postoperative length of hospitalization: assessed up to 90 days, rate of complications after discharge: up to 5 years after surgery
"Clinical Efficacy Between Robotic and Laparoscopic Total Gastrectomy in Patients With Clinical Stage I Gastric Cancer" (NCT03524300) [38]; Fujian Medical University; Country: China; Expected year of completion: 2024	Randomized Controlled Trial	Purpose of this study is to explore the clinical efficacy between robotic and laparoscopic total gastrectomy in patients with clinical stage I gastric cancer	220 patients, Experimental: Robotic Assisted Total Gastrectomy, Active Comparator: Laparoscopic Assisted Total Gastrectomy	Three-year disease-free survival rate	Three-year overall survival, three-year recurrence rate, overall postoperative morbidity rates: 30 days, intraoperative morbidity rate, overall post-operative serious morbidity rates; Clavien-Dindo IIIA or higher: 30 days, time to first ambulation: hours, time to first flatus, time to first liquid intake, time to first soft intake, duration of hospitalization, the variation of weight, the variation of white cell count, hospitalization expenses, operative time,
“Prospective Comparison of Surgical Outcomes With Using Integrated Robotic Technology Versus Conventional Laparoscopy for Gastric Cancer Surgery” (NCT03396354); Yonsei University; Country: South Korea; Expected year of completion: 2023	Non-randomized clinical trial	Investigate the number of harvested lymph nodes, using integrated robotic technology	140 patients, experimental arm: RG using Firefly or single site technology, comparator: laparoscopic surgery,	Number of retrieved lymph nodes	Blood loss, 3-year recurrence-free survival, 5-year overall survival, Overall complication rates, and amylase/lipase levels will be measured as markers of pancreatic parenchyma damage, CRP will be measured up to 4 weeks after surgery: inflammation, Pain scores up to 72 hours post-surgery, Wound complications

## Review

Postoperative outcomes

Length of Hospitalization 

The duration of hospitalization was a prominent parameter of investigation across the reviewed literature, with a total of 24 studies exploring it. Among these, nine studies provided evidence supporting the use of the robotic approach over the laparoscopic, after observing a statistically significant reduction in the length of hospitalization [5,12,14,15,26,27,32,34,35]. In contrast, 15 studies reported no statistically significant differences between the two minimally invasive approaches [9-13,16,17,19-21,24,25,29-31]. However, none of the reviewed studies favored the laparoscopic approach over the robotic. Yu et al. observed in more detail that the robotic approach was associated with a reduction in the total length of hospital stay by 0.81 days [8]. Chuan et al. concluded a substantial decrease of approximately two days in the length of hospital stay with robotic gastrectomy, from -3.66 to -0.3 days, when compared to laparoscopic gastrectomy [14]. Furthermore, Baral et al. observed a mean difference of -0.54 days in favor of the robotic approach [32], while Loureiro et al. reported a mean difference of -0.5 days between robotic gastrectomy and laparoscopic gastrectomy [34].

Conversion to Open Surgery 

In total, 13 studies investigated the rate of conversion of the robotic and laparoscopic approaches to open surgery. All of them concluded that the rate of conversion was relatively low for both approaches. For instance, Ojima et al. concluded that the conversion for the robotic approach was 3.4% versus 1.6% for the laparoscopic approach [3]. However, no statistically significant differences were observed in all the studies [3,5,7,10,11,15,22,24,26,29,32-34]

Time to First Flatus

The evaluation of time to first flatus in robotic gastrectomy versus laparoscopic gastrectomy revealed a range of findings among the 15 studies that examined it. Overall, nine out of the 15 studies reported a statistically significant decrease in time to the first flatus, in favor of the robotic approach [5,7,8,23,15,26,29,30,34]. Notably, Bobo et al. [7] concluded that the time to first postoperative flatus was 0.14 days shorter for the robotic group, with Jin et al. [29] and Yu et al. [8] further backing up this claim (-0,105 and -0.16 days, respectively). However, six studies found no statistically significant differences [10,22,24,27,31,32]

Time to Oral Intake

Regarding the time required to initiate oral intake, a clear trend across the eleven studies is evident. Ten studies [5,8,10,17,24,26,27,30,32,34] concluded that the patients who underwent robotic gastrectomy initiated oral intake earlier than those in the laparoscopic cohorts. Particularly, Guerrini et al. reported that the robotic group required 4.25 days to initiate oral intake, while the laparoscopic required 4.45 days [24]. Overall, the weighted mean difference ranged from -0.13 days [30] to -0.46 days [8] in favor of the robotic gastrectomy. Only Cong et al. found a non-statistical significant difference [31]. However, it should be noted that Sun et al. reported a different interval for liquid and soft diets [30]. They concluded that the robotic approach required less time for oral liquid intake, but no statistically significant difference was observed when comparing the time to oral-soft food intake.

Initiation of Adjuvant Chemotherapy, Postoperative Recovery, and Inflammatory Responses

Guerrini et al. [24] and Lu et al. [23] examined those parameters and they observed that the robotic group had an average quicker initiation of adjuvant chemotherapy by four days, quicker overall recovery, and milder inflammatory responses, when compared to the laparoscopic approach.

Reoperation Rate

Whilst analyzing the reoperation rates in six studies comparing robotic and laparoscopic gastrectomy, a stable trend emerges. It is evident that (a) the reoperation rate is very low in both approaches, notably 1.29% in the robotic and 1.23% in the laparoscopic [24], and (b) the differences in the reoperation rates are not statistically significant [5,7,24,26,29,34]. These results provide reassurance about the overall efficacy and safety of both approaches; however, the number of studies that compared the reoperation rates was only six, substantially lesser compared to studies that compared other outcomes (i.e.) blood loss, operative time, etc.

Overall Postoperative Morbidity 

In total, only four studies were identified in the literature that examined the overall post-operative morbidity. Lu et al. [23] observed an overall postoperative morbidity of 9.2% for the robotic approach and 17.6% for the laparoscopic, with Guerrini et al. [24] backing up this outcome. However, Xiong et al. [9] and Xiong et al. [10] did not find a statistically significant difference in the overall postoperative morbidity between the two approaches.

Complications 

Overall and Surgical Complication Rates

Twenty-two studies reported the overall complication rates, out of which three studies [5,26,29], reported a statistically significant decreased rate of overall complication in favor of the robotic approach, and 19 reported no statistically significant differences between the two approaches [7,8,11-17,19,21,23,24,27,30-34]. Notably, none of the studies seem to favor the laparoscopic over the robotic approach in terms of decreasing the overall complication rate.

Interestingly, five studies reported surgical complications using the Clavien-Dindo scale (CD) [3,24,30,33,34]. Sun et al. reported the surgical outcomes for the CD grades I-II and III or higher, which concluded that there was not a statistically significant difference between the two approaches [30]. However, Ojima et al. observed that the robotic approach had a statistically significant lower rate of complication regarding CD II or higher grades (8.8% vs 19.7%, robotic and laparoscopic approach, respectively) [3]. Similar outcomes were observed in the same study regarding CD III grade or higher, with the robotic approach having a complication rate of 5.3%, whereas the laparoscopic approach reached 19.7%. The results of Ojima et al. [3] agree with the results from Loureiro et al. [34], Ali et al. [33], and Guerrini et al. [24], which observed that the robotic approach decreased the surgical complication rate by 32%, 40%, and 34%, respectively, for CD III grades or higher.

Nine studies reported the rate of anastomotic leakage between the two approaches; however, none reached statistical significance [5,10,11,24,26,27,30,33,34]. Moreover, one study reported the outcomes for duodenal stamp leakage [26], one for anastomosis stenosis [21], two for intestinal obstruction rate [10,11], and two for ileus [26,30], but none of them reached statistical significance. Regarding delayed gastric emptying, three studies that examined this parameter found no statistically significant differences [5,27,30]. In addition, one study examined the wound complications and determined that there was not a statistically significant correlation between the surgical approaches [30].

Pancreatic Complications

Five studies examined the overall pancreatic complications, three of which reported statistically significant outcomes in favor of the robotic approach [5,29,34] and two presented non-statistical significant outcomes [20,24]. Particularly, Jin et al. [29] and Loureiro et al. [34] reported that the robotic approach can decrease the overall rate of pancreatic complications by 62.4% and 49%, respectively, when compared to the laparoscopic approach., Regarding postoperative acute pancreatitis, only Guerra et al. reported the outcomes and concluded a non-statistical significant relationship when comparing the surgical approaches [20]. Similarly, Zheng et al. [27] and Guerra et al. [20] reported a non-statistical relationship between the surgical approaches and the rate of postoperative pancreatic fistula. On the contrary, Cong et al. observed a statistically significant decline, by 67%, in the rate of postoperative pancreatic fistula formation in the robotic cohort when compared to the laparoscopic one [31].

*Intra-Abdominal Infections* 

Two studies examined the relationship between the surgical approach and the rate of intra-abdominal infections [3,30]. Ojima et al. concluded that the rate of intra-abdominal infections was slightly lower for the robotic approach when compared to the laparoscopic (6.2% vs 8.5%, robotic gastrectomy vs. laparoscopic gastrectomy, respectively); however, the relationship did not reach statistical significance [3]. Similarly, Sun et al. did not achieve statistically significant results either [30].

Surgical efficiency and costs

Operative Time

Robotic gastrectomy requires more operative time when compared to laparoscopic gastrectomy. In total, 25 studies investigated the time required to complete the gastrectomy, with either approach and all of them concluded a statistically significant increased operative time for robotic gastrectomy [5,7-17,19,20,22,24-27,29-32,34,35]. Notably, the average operative time was 267.34 minutes for the robotic approach and 220.48 minutes for the laparoscopic [24]. Similar results regarding the operative time were observed by Baral et al. [32]. Lastly, the average increase in the surgical duration for the robotic approach varied, ranging from 21.49 minutes [16] to 68.77 minutes [9], when compared to the laparoscopic approach.

Blood Loss

The analysis of blood loss in studies comparing robotic gastrectomy and laparoscopic gastrectomy reveals a consistent trend with very few variations. In total, 26 studies investigated blood loss, with 22 studies consistently reporting that the robotic approach had resulted in statistically significant lower blood loss when compared to the laparoscopic approach [5,7,9-11,13-15,17,19,22-24,26,27,29-35]. Overall, the mean intraoperative blood loss was 98.77 cc for the robotic and 115.07 for the laparoscopic approach [24], with the weighted mean difference ranging from approximately -42 mL [11] to -16 mL [14], in favor of robotic gastrectomy. However, it is important to underline that a few studies did not find a statistically significant difference in blood loss between the robotic and the laparoscopic approaches [8,12,16,26].

Costs

Regarding the differences in the costs, a clear trend emerges from the seven studies that examined this parameter: robotic gastrectomy is more expensive than the laparoscopic approach [5,24-26,29,34,35]. Notably, Guerrini et al. observed that the average cost for robotic gastrectomy was $12,224.54, whereas the laparoscopic approach totaled an average of $8,292.78 [24]. Similar trends were seen by Jin et al. [29], who concluded a weighted mean increased difference of 19,141.68 (Chinese Yuan), and Loureiro et al. [34] noted a mean difference of $3,684.27 in the robotic approach when compared to the laparoscopic. It should be noted, however, that Lu et al. concluded that the direct (rather than the total costs) were lower for the robotic cohort [25].

Mortality, survival, recurrence rate, recurrence, and disease-free survival

Mortality Rates

The assessment of mortality rates in laparoscopic and robotic gastrectomy procedures, reveals a consistent trend across all studies that examined these parameters. Specifically, the data from 15 studies indicate that there is no statistically significant differences in mortality rates between the laparoscopic and robotic approaches [5,7,9-11,15,21-23,24,27,29,32,34,35].

Furthermore, the mortality rates observed in both approaches are remarkably low, particularly 0.36% for the robotic and 0.30% for the laparoscopic approach [24]. Whilst precise data may exhibit slight variations across different studies, the overall pattern firmly attests to the safety of both the robotic and laparoscopic approaches.

Recurrence Rate 

The analysis of recurrence rates between the two surgical approaches reveals that both approaches have similar recurrence rates with no statistically significant differences. Specifically, Guerrini et al. observed a recurrence rate of 9.9% for the robotic gastrectomy group compared to 13.5% for the laparoscopic gastrectomy group [24]. Similarly, Wu et al. [28] observed a decrease of 12% in the recurrence rate in favor of the robotic approach. However, all eight studies that examined this parameter concluded non-statistically significant results [5,11,18,24,25,27,28,34].

Recurrence-Free Survival, Disease-Free Survival, and Overall Survival

Regarding recurrence-free survival, all four studies that examined this parameter determined that there was no statistically significant outcome when comparing the two approaches [5,11,25,26]. Similar findings were observed for disease-free survival by Liao et al. [11] and Pan et al. [18], who concluded non-statistically significant outcomes. In addition, the findings by Wu et al. further support the lack of significant differences between the two approaches, as their study concluded no difference in disease-free survival for both three-year and five-year postoperative periods [28]. Lastly, regarding overall survival, none of the eight studies examining this parameter found statistically significant differences between the approaches [5,11,18,23,26,28,29,32].

Pathology 

Distal Resection Margin

The distal resection margin, which refers to the length of healthy tissue removed during resection along with the tumor, was examined in 17 studies comparing robotic with laparoscopic approaches. The overall trend suggests that both approaches had similar distal resection margins (6.70 vs 6.49, robotic laparoscopic, respectively), with no statistically significant differences being observed in 14 studies [5,7,10,12,14,15,19,23,24,26,31-34].

Only two studies [13,27] found a significant increase in the distal resection margins for the robotic approach and Xiong et al. reported a significant decrease in the distal resection margins for the robotic approach when compared to the laparoscopic [10]. It should be noted that in the systematic review by Zizzo et al. [5], one study observed a significant increase in the resection margins, in favor of the laparoscopic approach and one in favor of the robotic approach.

*Proximal Resection Margin* 

The assessment of the proximal resection margins in the robotic approach versus the laparoscopic, reveals a consistent and stable trend across 16 studies that examined this parameter. Specifically, both approaches exhibited similar proximal resection margins, i.e. 4.46 cm for the robotic and 4.35 cm for the laparoscopic [24]. However, none of the studies observed any statistically significant differences [5,7,10-15,23,24,26,27,31-34].

Number of Lymph Nodes

The number of harvested lymph nodes was one of the most studied parameters in the literature. However, the results are inconclusive, as out of the 23 studies, 13 found a statistically significant increase in the number of harvested lymph nodes in favor of the robotic approach [5,15,20,24-27,29,30-34], whereas 10 found no statistically significant relationship between the robotic and the laparoscopic approach [7,9,11-14,16,17,19,25].

Notably, Guerrini et al. observed that the RG group harvested approximately 1.5 more lymph nodes when compared the the LG group [24]. Similarly, Lu et al. [25] reported that the RG group had approximately 2 more harvested extra-gastric lymph nodes. Guerra et al. [20] and Jin et al. [29], observed a higher mean number of 2.92 and 2.03, respectively, harvested lymph nodes in the robotic gastrectomy group compared to laparoscopic gastrectomy. Lastly, none of the studies showed a significant increase in the number of harvested lymph nodes, in favor of the laparoscopic approach.

Clinical trials 

A study titled “CLASS14” by Qingdao University, China, (NCT06042998), with an expected date of completion in 2027, aims to investigate the clinical efficacy of the robotic radical gastrectomy in patients with gastric adenocarcinoma (cT2N+M0 or cT3-4a/N+M0), undergoing neoadjuvant chemotherapy and comparing it to the conventional laparoscopic approach [36]. In total, 294 patients will be allocated in each group. The main outcome of the study is the three-year disease-free survival with the secondary outcomes being the three-year overall survival, the overall postoperative morbidity rate, and the 14-day postoperative recovery course. This clinical trial is of particular importance as the three-year disease-free survival is currently underrepresented in the literature. Particularly, only Liao et al. [11], Pan et al. [18], and Wu et al. [28] examined this parameter and all three did not achieve statistically significant differences. Furthermore, the overall postoperative morbidity rate is also underrepresented in the literature and the four studies that examined it resulted in conflicting evidence. Specifically, Lu et al. [25] and Guerrini et al. [24] concluded that the robotic approach significantly decreased the overall postoperative morbidity rate, but Xiong et al. [13] and Xiong et al. [9] did not observe a statistically significant difference. This clinical trial can shed light on whether the robotic approach actually decreases the overall postoperative morbidity rate or whether the differences are not significant. The results can support surgical teams in deciding whether the postoperative morbidity rate is a factor that should be taken into consideration when choosing between the robotic and the laparoscopic approach.

A prospective, observational multicenter study, namely the IMIGASTRICII study, from the international study group on minimally invasive surgery for gastric cancer (NCT02751086) [37], aims to create a multi-institutional database of data regarding the clinical, surgical, and oncological features of patients who undergo robotic, laparoscopic, or open gastrectomy. The study aims to include 50,000 patients in total and it is expected to be completed in 2024. The study will primarily investigate the number of harvested lymph nodes and the mortality rate and secondarily the mean length of hospitalization and the rate of complications. Even though the number of harvested lymph nodes, the mortality rate, and the length of hospitalization have been highly investigated before, the results from the postoperative complications are of particular interest as the rates of abdominal infections, duodenal stamp leakage, anastomosis stenosis, intestinal obstruction, ileus, delayed gastric emptying, wound complications, acute pancreatitis, and pancreatic fistula are underreported and often overlooked in the literature. This study could potentially shed light on less commonly anticipated complications and to an extent assist surgical teams in determining the best surgical approach for their patients.

A randomized clinical trial, titled "Clinical efficacy between the robotic and laparoscopic total gastrectomy in patients with clinical stage I gastric cancer”, from the Fujian Medical University in China (NCT03524300) aims to investigate the clinical efficacy of robotic and laparoscopic surgery for stage I gastric cancer [38]. In total, 220 patients will be split into two groups, namely the robotic and the laparoscopic group and the researchers will measure primarily the three-year disease-free survival. Secondary outcomes include the three-year overall survival, three-year recurrence rate, overall postoperative morbidity rates (30 days), intraoperative morbidity rate, overall post-operative serious morbidity rates; Clavien-Dindo IIIA or higher (30 days), time to first ambulation, time to first flatus, time to first liquid intake, time to first soft intake, duration of hospitalization, the variation of weight, the variation of white cell count and the hospitalization expenses. This study is of particular importance as it will examine the overall post-operative and intra-operative morbidity rates. Currently, four studies [9,10,24,25] reported overall morbidity with conflicting evidence. Moreover, this study makes a clear distinction between the time to first liquid or soft intake rather than reporting it as an overall oral intake. It is noteworthy, that Sun et al. were the only ones who made that distinction and concluded that the robotic approach required less time for oral liquid intake, but a statistically significant difference was not observed for soft food intake [30]. Moreover, the variation in white cell count will also be measured as an indication of inflammation. Inflammatory responses are underreported in the literature and so far, only Lu et al. [25] and Guerrini et al. [24] were found to be reporting the inflammatory responses. They concluded that the robotic approach had milder inflammatory responses compared to the laparoscopic; however, more research is needed to support this claim. Hospitalization expense is another parameter examined by this clinical trial. From the literature, it is clear that the robotic approach is far more expensive than the laparoscopic, however, it would be interesting to analyze whether the direct costs are actually lower for the robotic compared to the laparoscopic, as shown by Lu et al. [25]. Lastly, the variation in weight will be measured as no other studies included in this literature review reported it. It would be worth delving into how weight variation following gastrectomy for gastric cancer can influence the quality of life.

A non-randomized clinical trial, titled "Prospective comparison of surgical outcomes", by the Yonsei University in South Korea (NCT03396354), aims to investigate the number of harvested lymph nodes using integrated robotic technology [39]. In total, 140 patients will be involved in two arms: the experimental group which will take place using Firefly or single-site technology, and the conventional laparoscopic approach. Apart from the number of harvested lymph nodes, the study will secondarily measure the total blood loss, the three-year recurrence-free survival, the five-year overall survival, overall complication rates, and amylase/lipase as markers of pancreatic parenchyma damage. C-reactive protein (CRP) will also be measured up to four weeks after surgery. Lastly, pain scores up to 72 hours post surgery and the rate of wound complications will be recorded too. The recurrence-free survival rate is currently underreported in the literature, with only four studies reporting this outcome [5,11,25,26] and they all concluded a non-statistical significant relationship when comparing the two approaches. It will be intriguing to see whether this study will produce any different outcomes. Also, the study will measure the pancreatic parenchyma damage via amylase/lipase sampling. The study can possibly outline whether the degree of amylase/lipase can predict pancreatic complications, such as pancreatic fistula formation and acute pancreatitis, currently underreported in the literature. Moreover, the study will examine the CRP as an indication of inflammation. So far only Lu et al. [25] and Guerrini et al. [24], were found to be reporting the inflammatory responses and they concluded that the robotic approach had milder inflammatory responses compared to the laparoscopic. Wound complications will also be examined and provide further evidence to support or challenge the findings of Sun et al., who found no statistically significant relationship between the surgical approach and the rate of wound complications [30]. Lastly, pain levels will be examined, which have not been extensively reported in the literature.

## Conclusions

The robotic approach was statistically significantly associated with a decreased length of hospitalization, overall complication rates, surgical complications (Clavien-Dindo), pancreatic complications, blood loss, time to first flatus, and initiation of oral intake. Also, it was associated with an increased number of harvested lymph nodes, in the majority of the studies. However, it was associated with increased operative duration and costs. No major differences were observed in conversion to open surgery, anastomotic leakage, mortality rate, distal and proximal resection margins, recurrence rate, reoperation rates, and overall survival. Lastly, the rate of abdominal infections, duodenal stamp leakage, anastomosis stenosis, intestinal obstruction, ileus, delayed gastric emptying, wound complications, acute pancreatitis, pancreatic fistula, direct costs, time to initiate adjuvant chemotherapy, overall postoperative morbidity, recurrence-free survival, and disease-free survival were underreported in the literature and necessitate further research. Overall, the robotic approach has equal if not superior oncological, short, and long-term outcomes compared to the laparoscopic approach. However, there are still plenty underreported outcomes that necessitate further RCTs.
